# Analysis of Changes in Electrolytes Level in Serum After Death and Its Correlation With Postmortem Interval

**DOI:** 10.7759/cureus.38957

**Published:** 2023-05-13

**Authors:** Aravindan Umapathi, Hitesh Chawla, Sangeeta B Singh, Ashish Tyagi

**Affiliations:** 1 Forensic Medicine, Shaheed Hasan Khan Mewati Government Medical College, Nalhar, Nuh, IND; 2 Biochemistry, Shaheed Hasan Khan Mewati Government Medical College, Nalhar, Nuh, IND

**Keywords:** potassium, sodium, postmortem interval, time since death, electrolytes, serum, thanatochemistry

## Abstract

Background

Apropos estimation of postmortem interval is an important and difficult task for forensic pathologists. In routine practice, postmortem interval is deduced by conventional or physical methods such as early and late postmortem changes, which are subjective methods and prone to errors. Estimating time since death by thanatochemistry is a more objective method as compared to routine conventional or physical methods. The present study is an attempt to analyze the changes in electrolytes level in serum after death and its correlation with postmortem interval.

Materials and methods

Blood samples were taken from the deceased who were brought for a medicolegal autopsy. The concentration of electrolytes, mainly sodium, potassium, calcium, and phosphate, was evaluated in the serum. The deceased were grouped on the basis of time since death. Log-transferred regression analysis was done to establish the correlation of the concentration of electrolytes with time since death and regression formulas were derived for each parameter.

Results

Sodium concentration in serum showed a negative correlation with time since death. Potassium, calcium, and phosphate showed a positive correlation with time since death. No statistically significant difference exists in the concentration of electrolytes between males and females. No significant difference was observed in the electrolytes concentration between the age groups.

Conclusion

Considering the findings of this study, we infer that the concentration of electrolytes, primarily sodium, potassium, and phosphates, in the blood can be used to approximate the amount of time that has passed since death. Nonetheless, until 48 hours after death, electrolyte levels in the blood can be considered for the calculation of the postmortem interval.

## Introduction

Postmortem interval (PMI) is the period between a person's death and the discovery of their body [1]. Determination of the postmortem interval (PMI) or time since death (TSD) is an essential element of a medicolegal autopsy [2]. A precise estimation of time since death can verify the statements of the witness of the crime and may limit the number of possible suspects related to the crime [3]. It is challenging for a forensic pathologist to estimate the time since death precisely. In routine, early and late postmortem changes over the body are employed for an estimate of the time since death. However, these early and late postmortem changes over the body are strongly influenced by numerous internal and external factors such as ambient temperature, the humidity in the air, clothing, the age of the deceased, the weight of the body, etc. Therefore, the actual time of death can be hardly estimated based on routine physical or conventional methods [4,5]. The recent developments of biochemical methods for the estimation of time since death have entirely changed the face of the evaluation of postmortem interval. The biochemical methods are more sensitive, systematic, and less prone to errors as compared to routine physical methods of time since death estimation [6]. Using postmortem chemistry has become one of the most important supplemental techniques for forensic pathologists. Estimation of postmortem interval from forensic biochemistry is a type of quantitative test. It is said to be a more objective method as compared to all the other existing methods. Biochemical changes can be observed soon after death in bodily fluids such as blood, pericardial fluid, cerebrospinal fluid (CSF), synovial fluid, and vitreous humor. These changes progress sequentially until the body fully disintegrates [7,8]. From the forensic viewpoint, the foremost advantage of the blood is that it is chiefly distributed all over the body and not readily affected by confounding factors like age, gender, diet, diurnal cycles, and stress, making it ideal for estimating time since death [9]. Peculiar degenerative changes take place in the blood and its components after death, predominantly immediately after somatic death, as per the literature available. By using this principle, the postmortem interval can be divined successfully in day-to-day forensic practice where the time since death is indeterminate [10].

Numerous studies have been conducted in the past on biochemical changes in the vitreous humor, synovial fluid, and cerebrospinal fluid after death. However, only limited literature is available on postmortem biochemistry on serum and time since death. Blood has a major advantage as it is homogeneous, widely distributed in the body, and easily accessible during autopsy making it less liable to be contaminated during extraction. The present study is therefore contemplated to study the changes in the level of the electrolytes, namely sodium, potassium, calcium, and phosphate in serum after death and its correlation with postmortem interval.

## Materials and methods

The cross-sectional study was conducted in the Department of Forensic Medicine in collaboration with the Department of Biochemistry at a tertiary care center in Southern Haryana, India. The approval of the Institutional Ethics Committee (IEC) was taken prior to the initiation of the study (approval letter no. EC/OA-11/2021). The medicolegal deaths with known time since death and the deaths which were declared by a doctor were included in the study during the period ranging from April 2021 to March 2022. The cases with unknown time since death, hemolyzed postmortem blood samples, or where the process of putrefaction has started already were excluded from the study. Deceased with a history of medications that might alter the electrolyte concentration were also excluded from the study. The history was obtained from the accompanied relatives of the deceased. After obtaining informed written consent from the relatives or legal heir of the deceased, the blood samples were collected before proceeding with the dissection of the dead body. As compared to other peripheral veins of the body, the external jugular vein (EJV) is a prominently visible superficial vein that also delivers a considerable amount of blood for examination. Therefore, the blood was collected from the external jugular vein.

Sample collection and processing

With the dead body lying supine and the head turned to one side in the neutral plane, the blood was collected from EJV under aseptic precautions [11]. The sample collected was sent to the biochemistry laboratory without any delay. Samples were then centrifuged at 3,000 revolutions per minute (RPM) for three to four minutes to segregate serum from the cellular parts. The supernatant fluid, serum, was used for analysis.

The ROCHE Electrolyte Analyzer was used to calculate sodium and potassium levels in the serum by the ion-selective electrode (ISE) method. Spectrophotometric calculations were used by the completely automated analyzer (ROCHE COBAS c501) to assess the calcium and phosphate levels. The reports were collected from the Department of Biochemistry on the same day.

Statistical analysis

The collected data was further analyzed with IBM SPSS Statistics for Windows, Version 23.0. (Armonk, NY: IBM Corp). For categorical variables, percentage analysis was used to explain the data frequency analysis, while mean and standard deviation (SD) were employed for continuous variables. An unpaired sample t-test was performed to determine the significance of the difference between the bivariate samples for independent groups. Pearson's correlation was utilized to determine the relationship between the variables, and the scatter plot was employed to display the results. Log-transformed regression analysis was utilized to predict the PMI model. In each of the aforementioned statistical methods, a probability value of less than 0.05 was deemed to be statistically significant.

## Results

A total of 100 samples of serum were studied for electrolyte concentration during the study period. The mean age of the study subjects was 31.1 ± 16.64 years. Based on the postmortem interval, the subjects were divided into seven groups (Table 1).

**Table 1:** Distribution of cases based on their postmortem interval PMI: postmortem interval.

PMI in hours	Frequency
≤ 6 hours	6
6.1-12 hours	18
12.1-18 hours	19
18.1-24 hours	32
24.1-36 hours	17
36.1-48 hours	6
> 48 hours	2
Total	100

The majority (32%) falls under the PMI group of 18.1 to 24 hours. The minimum and maximum postmortem interval was 2.5 hours and 52 hours, respectively. The descriptive statistics of serum sodium, potassium, calcium, and phosphate concentration with PMI are depicted in Table 2.

**Table 2:** Serum sodium, potassium, calcium, and phosphate concentration in relation to PMI PMI: postmortem interval.

PMI (hours)	N	Mean ± SD of sodium concentration (mEq/L)	Difference in mean sodium concentration (mEq/L)	Mean ± SD of potassium concentration (mEq/L)	Difference in mean potassium concentration (mEq/L)	Mean ± SD of calcium concentration (mg/dL)	Difference in mean	Mean ± SD of phosphate concentration (mg/dL)	Difference in mean
≤ 6 hours	6	138.33±2.42	---	16.65±2.23	---	6.23±0.54	---	20.18±2.55	---
6.1-12 hours	18	126.06±11.56	-12.28	25.10±11.99	8.45	7.52±1.65	1.29	29.33±8.44	9.15
12.1-18 hours	19	110.16±15.74	-15.90	33.45±8.57	8.35	9.28±1.68	1.76	34.21±5.80	4.88
18.1-24 hours	32	111.44±10.95	1.28	36.89±7.19	3.44	9.70±4.94	0.42	36.00±6.63	1.79
24.1-36 hours	17	109.00±8.18	-2.44	38.17±4.31	1.28	14.30±16.28	4.60	37.36±6.11	1.37
36.1-48 hours	6	91.6±4.84	-17.33	32.50±11.39	-5.67	6.30±1.96	-8.00	41.27±10.15	3.90
> 48 hours	2	113.50±27.58	21.83	22.70±0.00	-9.80	11.15±1.48	4.85	32.70±5.23	-8.57
Mean PMI 19.45 hours	100	113.88±15.07	Mean rate of decrease: 1.34 mEq/L/h	32.569±10.15	Mean rate of increase: 1.02 mEq/L/h	9.62±7.59	Variable	33.99±8.11	Mean rate of increase: 1.5 mg/dL/h

Serum sodium concentration in relation to PMI

The mean sodium was decreasing from 138.33 mEq/L in PMI of ≤ 6 hours to 110.16 mEq/L in PMI of 12.1-18 hours. A slight rise in mean sodium concentration was observed in the PMI group of 18.1-24 hours (111.44 mEq/L), after which it further falls till 48 hours (91.67 mEq/L) of PMI. After 48 hours of PMI, sodium concentration in the serum starts rising again. The rate of decrease in mean sodium concentration is 1.34 mEq/L per hour.

Serum potassium concentration in relation to PMI

The mean potassium concentration was increasing from 16.65 mEq/L at ≤ 6 hours of PMI to 38.17 mEq/L at 24.1-36 hours of PMI. The potassium concentration started decreasing after 36 hours of PMI.

Serum calcium concentration in relation to PMI

The mean calcium concentration was continuously increasing from 6.39 mg/dL at a PMI of ≤ 6 hours to 14.30 mg/dL at a PMI of 24.1-36 hours. A gross decrease in mean calcium level was observed at a postmortem interval of 36.1-48 hours (6.30 mg/dL). An increase in mean calcium concentration (11.15 mg/dL) was observed, after 48 hours of postmortem interval.

Serum phosphate concentration in relation to PMI

The mean phosphate concentration in the serum was constantly rising from 20.18 mg/dL at a PMI of ≤ 6 hours to 41.27 mg/dL at a PMI of 36.1-48 hours. A gross decline of mean phosphate concentration was observed at postmortem interval of > 48 hours (32.70 mg/dL). The rate of increase in mean phosphate concentration is 1.5 mg/dL per hour.

Log-transformed regression analysis

**Figure 1:**
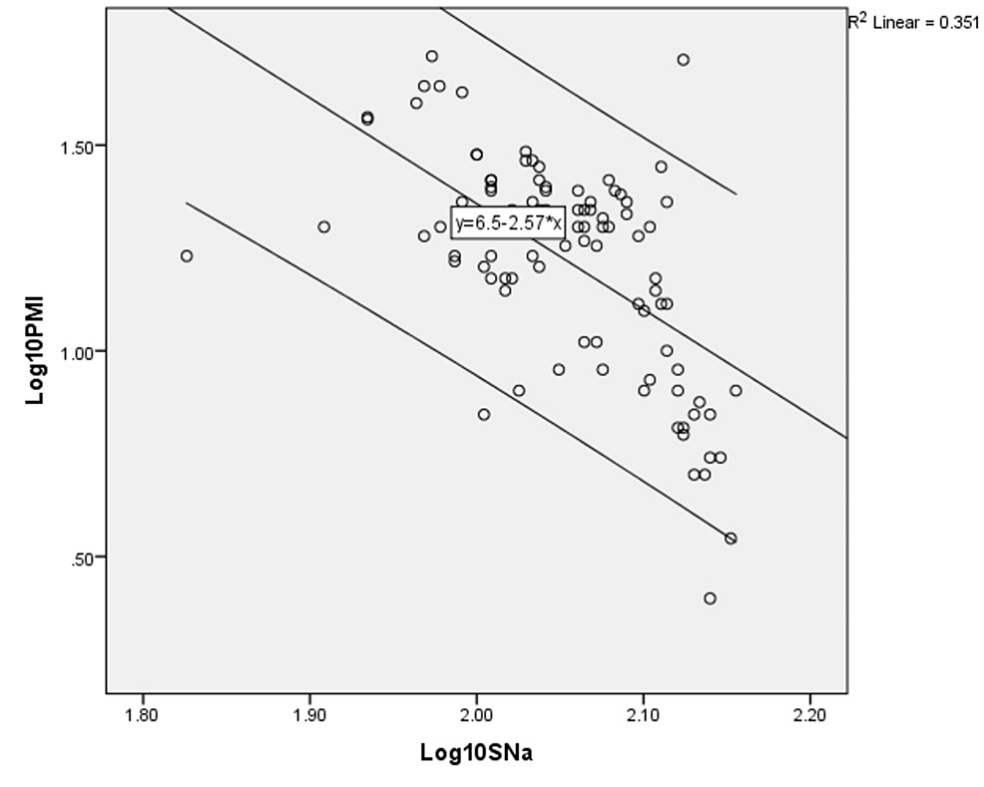
The scatter plot shows a negative correlation between Log10 serum sodium concentration with Log10 PMI with R2 = 0.351 with 95% C.I which is parallel to the regression line PMI: postmortem interval.

**Figure 2:**
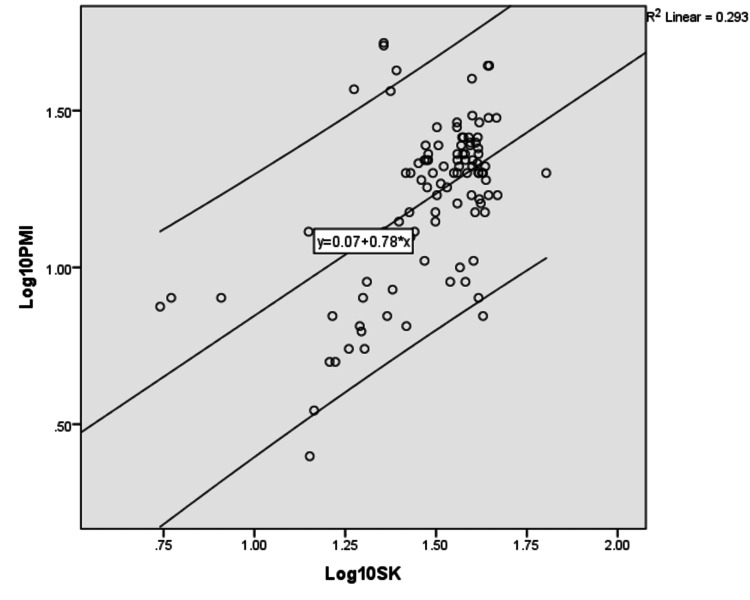
The scatter plot shows a positive correlation between Log10 serum potassium concentration with Log10 PMI with R2 = 0.293 with 95% C.I which is parallel to the regression line PMI: postmortem interval.

**Figure 3:**
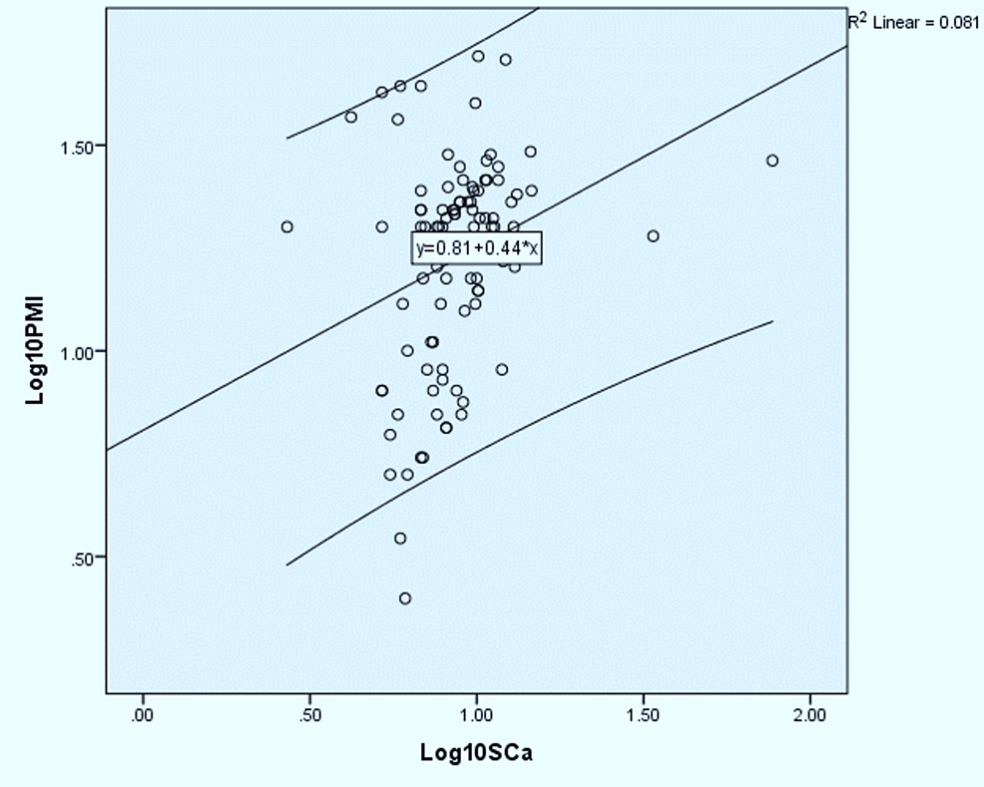
The scatter plot shows a positive correlation between Log10 serum calcium concentration with Log10 PMI with R2 = 0.081 with 95% C.I which is parallel to the regression line PMI: postmortem interval.

**Figure 4:**
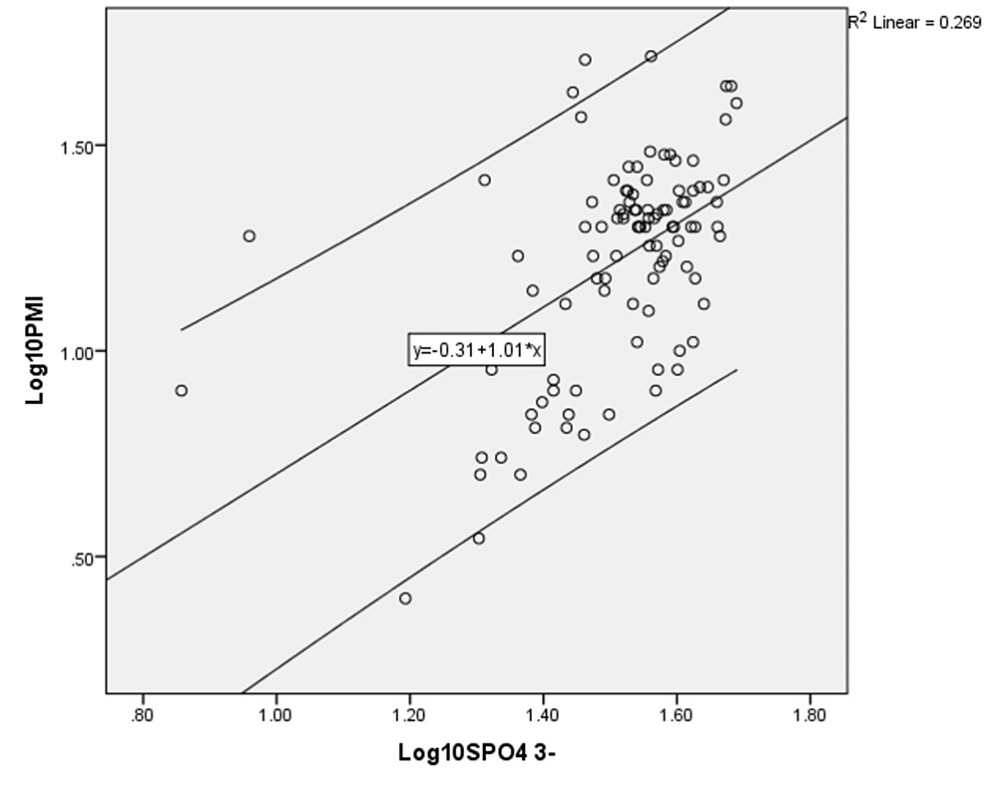
The scatter plot shows a positive correlation between Log10 serum phosphate concentration with Log10 PMI with R2 = 0.269 with 95% C.I which is parallel to the regression line PMI: postmortem interval.

The regression formulas were derived for individual parameters. The log-transformed linear regression analysis performed to correlate serum sodium, potassium, calcium, and phosphate concentration with postmortem interval is depicted in Tables 3-6 and their scatter diagram plot is presented in Figures 1-4. All the derived regressions were statistically highly significant with a p-value of 0.0005 (<0.01).

**Table 3:** Log10 serum sodium concentration with Log10 PMI by log-transformed linear regression **Highly statistical significance at p < 0.01 level. PMI: postmortem interval, LB: lower bound, UB: upper bound.

Model	Unstandardized coefficients		Standardized coefficients	t	p-value	95.0% C.I for B		R	R^2^
	B	Standard error	Beta			LB	UB		
Constant	6.498	0.725		8.967	0.000	5.060	7.936	0.593	0.351
Log_10 _serum Na	-2.570	0.353	-0.593	-7.283	0.0005**	-3.271	-1.870		

**Table 4:** Log10 serum potassium concentration with Log10 PMI by log-transformed linear regression **Highly statistical significance at p < 0.01 level. PMI: postmortem interval, LB: lower bound, UB: upper bound.

Model	Unstandardized coefficients		Standardized coefficients	t	p-value	95.0% C.I for B		R	R^2^
	B	Standard error	Beta			LB	UB		
Constant	0.067	0.183		0.368	0.714	-0.295	0.430	0.541	0.293
Log_10 _serum K	0.779	0.122	0.541	6.369	0.0005**	0.536	1.022		

**Table 5:** Log10 serum calcium concentration with Log10 PMI by log-transformed linear regression **Highly statistical significance at p < 0.01 level. PMI: postmortem interval, LB: lower bound, UB: upper bound.

Model	Unstandardized coefficients		Standardized coefficients	t	p-value	95.0% C.I for B		R	R^2^
	B	Standard error	Beta			LB	UB		
Constant	0.807	0.144		5.613	0.000	0.522	1.092	0.284	0.081
Log_10 _serum Ca	0.443	0.151	0.284	2.936	0.0005**	0.143	0.742		

**Table 6:** Log10 serum phosphate concentration with Log10 PMI by log-transformed linear regression **Highly statistical significance at p < 0.01 level. PMI: postmortem interval, LB: lower bound, UB: upper bound.

Model	Unstandardized coefficients		Standardized coefficients	t	p-value	95.0% C.I for B		R	R^2^
	B	Standard error	Beta			LB	UB		
Constant	-.311	0.256		-1.214	0.228	-.820	0.197	0.519	0.269
Log_10 _serum PO_4_^3-^	1.013	0.169	0.519	6.004	0.0005**	0.678	1.347		

Log-transformed linear regression formulas to correlate postmortem interval with serum sodium, potassium, calcium, and phosphate are given below, respectively.

Log_10_ PMI = Log_10_ Serum Sodium × (-2.570) + 6.498

Log_10_ PMI = Log_10_ Serum Potassium × 0.779 + 0.067

Log_10_ PMI = Log_10_ Serum Calcium × 0.443 + 0.807

Log_10_ PMI = Log_10_ Serum Phosphate × 1.013 - 0.311

## Discussion

Several studies have been done on forensic biochemistry to estimate the time since death from various body fluids such as vitreous humor, CSF, blood, synovial fluid, and pericardial fluid. Variations in electrolyte concentration, enzyme level, protein content, pH, etc. have been analyzed to know whether the biochemical changes in those body fluids can be used to estimate the time since death or not. In the present study, electrolyte changes in serum namely sodium, potassium, calcium, and phosphate were analyzed to determine postmortem interval.

A negative correlation was observed between the concentration of sodium and the postmortem interval. The mean sodium concentration in serum was continuously decreased till 18 hours of PMI. A slight increase in mean serum sodium concentration at a PMI of 18.1 to 24 hours was observed. Thereafter, the sodium concentration was decreasing till 48 hours of PMI. There was further an increase in sodium concentration after 48 hours of PMI. Hodgkinson and Hambleton documented serum sodium falls rapidly after death [12]. Coe mentioned that the serum sodium concentration started falling immediately after death at the rate of 0.9 mEq/L per hour [7]. Singh et al. observed the serum sodium concentration decreased from 132.58±4.23 mEq/L to 109.91±4.35 from the postmortem interval of 3 hours to 58 hours [13]. The rate of fall in serum sodium concentration was observed to be 0.8 mEq/L per hour [13]. Das et al. observed in their study of 150 cases, in which an equal number of cases were distributed in every postmortem interval group, that the mean serum sodium concentration falls at a rate of 1.14 mEq/L per hour up to 33 hours of PMI [14]. As per the current study, the rate of fall in mean sodium concentration was observed to be 1.34 mEq/L per hour. The variation may be due to the different sample sizes in different studies. Previous studies have observed a consistent fall in serum sodium concentration after death. But in the current study, a slight rise in the mean serum sodium concentration was observed in the PMI group of 18.1 to 24 hours; however, it was only a slight rise. This may be due to the variation in the number of cases in each PMI group. But a drastic change in serum sodium concentration was observed after 48 hours of death, with sudden rise as compared to persistent fall till that time.

As the potassium begins to come out of the cell after death, the serum potassium concentration increased very rapidly after death. We observed that the concentration of potassium (mEq/L) in serum with PMI (hours) was continuously increasing till 36 hours of PMI, and there was a drop in the mean potassium concentration after 36 hours. Hodgkinson and Hambleton observed that the postmortem serum potassium concentration markedly increased after death [12]. Coe also observed that the serum potassium concentration increased up to 10 mEq/L within 15 minutes after death [7]. Singh et al. observed that the serum potassium concentration was increased after death from 13.95±4.77 mEq/L at PMI of 3-6 hours to 38.14±6.89 mEq/L at PMI of 48.1-58 hours. The mean rate of potassium rise was 1.02 mEq/L per hour after death [13]. All the previous researchers have mentioned a rise in serum potassium levels after death; in contrast, it was observed in the present study that the rise in potassium concentration was evident till 36 hours after death. After that, a drop in serum potassium was observed. This may be due to the small number of samples in the PMI group after 36 hours of death, or it may be due to the change in the composition of electrolytes as the time since death advances and putrefaction starts setting in.

The current study revealed that the concentration of calcium (mg/dL) in serum with PMI (hours) was continuously increased till 36 hours from 6.23 to 14.30 mg/dL and showed fluctuations thereafter. Hodgkinson and Hambleton mentioned that the serum calcium concentration did not change for the first four hours after death, but it started increasing 10 hours after death [12]. Fekete and Brunsdon in their work emphasized that postmortem serum calcium concentration elicited a wide range of variations and therefore not able to establish its correlation with time since death [15]. Coe also concluded that serum calcium elevates after death [7]. Zhu et al. in their research mentioned that there was no significant postmortem time-dependent rise in serum calcium [16]. The present study also observed the fluctuations in the mean change of calcium concentration in the serum. However, we observed a positive correlation between serum calcium concentration and time since death.

The concentration of phosphate (mg/dL) in serum with PMI (hours) was continuously increasing till 36.1-48 hours, from 20.18 mg/dL at PMI of ≤ 6 hours to 41.27 mg/dL at PMI of 36.1-48 hours in the present study. There was a drastic drop in the mean at > 48 hours. The rate of increase in mean phosphate concentration is 1.5 mg/dL per hour in the present study.

The results of the study on the electrolyte concentration in serum after death indicated that the change in electrolyte level, whether an increase or a decrease, is sustained for 36 hours or a maximum of 48 hours after death. After that, the trend of change shifted in the opposite direction. It showed that once the putrefaction sets in, which usually starts setting within 36 to 48 hours of death in this region, the electrolyte concentration cannot be taken into account for estimation time since death.

Limitations

Limitations in the study might be attributed mainly to the small sample size. Moreover, there was a disparity in the distribution of cases according to postmortem interval. The sample sizes for the different time intervals after death varied widely. In addition, the rate of decomposition might be affected by environmental and seasonal factors, and those factors were not taken into account. There was a lack of information on whether or not the deceased had electrolyte abnormalities during the antemortem period. The only source of information we had regarding the dead person's health and medications is the information provided by the accompanied relatives. Electrolyte abnormalities could be better ruled out if the premortem baseline level is used as a reference point.

## Conclusions

Forensic pathologists and law enforcement agencies throughout the world confront a number of pressing difficulties, one of which is determining an apt time of death. As perceived, this is essentially the purview of the forensic pathologist in conjunction with law enforcement agencies; nonetheless, it needs the combined efforts of an interprofessional team, comprising the forensic pathologist, law enforcement officials, as well as biochemists. Considering the findings of this study, we infer that the concentration of electrolytes, primarily sodium, potassium, and phosphates, in the blood can be used to approximate the amount of time that has passed since death. Nonetheless, until 48 hours after death, electrolyte levels in the blood can be considered for the calculation of the postmortem interval.
